# Cross-cultural validation of the attitudes toward research methods scale in teacher education: evidence from a Turkish sample including music teacher candidates

**DOI:** 10.3389/fpsyg.2026.1879775

**Published:** 2026-08-03

**Authors:** Cagri Sen, Zeynep Özer

**Affiliations:** 1 Department of Music, Faculty of Fine Arts and Design, Sinop University, Sinop, Türkiye; 2 Ministry of National Education, Bursa, Türkiye

**Keywords:** attitudes toward research methods, music teacher education, scale adaptation, teacher education, Turkish adaptation, validity and reliability

## Abstract

**Background:**

Attitudes toward research methods are important for teacher candidates’ research engagement, academic learning, and professional development. However, instruments that directly assess attitudes toward research methodology content have rarely been examined across cultural contexts.

**Aim:**

This study aimed to adapt the Attitudes towards Research Methods Questionnaire into Turkish and to examine the validity and reliability evidence of the adapted form in teacher education.

**Methods:**

The study was designed as a methodological scale adaptation study. Data were collected from 509 eligible pre-service teachers in Türkiye. After data screening, the main psychometric analyses were conducted with 486 cases. Linguistic equivalence and cultural fit were examined before the main study. Exploratory and confirmatory factor analyses were conducted on two independent randomly divided subsamples. Reliability, construct reliability, convergent validity evidence, item discrimination, criterion validity, and supplementary discipline-based comparisons were also examined.

**Results:**

The findings supported the three-factor structure of the original scale in the Turkish sample. The final 15-item form consisted of Negative Emotions, Utility, and Positive Emotions. The Utility factor reflected cognitive evaluations of research methods, whereas the Positive and Negative Emotions factors reflected affective components. The total Cronbach’s alpha and McDonald’s omega coefficients were both 0.90, supporting internal consistency. Item analyses and criterion validity findings also supported the psychometric adequacy of the scale. Supplementary comparisons showed no significant differences between music teacher candidates and candidates from other teacher education programs.

**Conclusion:**

The Turkish form of the *“Attitudes towards Research Methods Scale in Education”* provides valid and reliable scores for assessing attitudes toward research methods in teacher education contexts. Future studies should further test its validity in different cultural contexts and examine measurement invariance across groups.

## 1 Introduction

Educational research consists of systematic studies conducted to produce solutions to problems encountered within the education system and to improve existing practices (Çelen, 2023). These studies not only generate new knowledge but also contribute to the reinterpretation and development of learning, teaching, and educational management processes in line with current conditions (Gall et al., 2002; Mills and Jordan, 2023). In this context, today’s educational researchers need to be well-versed in different research approaches in order to understand complex educational problems and develop effective solutions (Creswell, 2012).

A recurring theme in the literature is that teachers are expected to demonstrate research competence within their professional practice and continuously follow recent developments in their disciplines (Ekinci and Ekinci, 2022; Insorio, 2024; Korkmaz et al., 2011; Uyar, 2024). Educators, often unaware of it, use inquiry, analysis, and evaluation processes in their professional decision-making, similar to scientific thinking (Kitsantas et al., 2023). Given these professional demands, research methods education represents a crucial component of teacher training programs (Kitsantas et al., 2023; Matos et al., 2023; Wishkoski et al., 2022). This training focuses on the procedures in designing, implementing, and analyzing educational research (Matas-Terrón et al., 2024).

In Türkiye, faculties of education include “Research Methods in Education” as a required component of teacher education programs, typically offered in the early years of undergraduate study (Uzun, 2020). This course is designed to introduce the stages of the research process, to provide conceptual knowledge about scientific research methods, and to enable students to develop skills such as literature review, data collection, analysis, and reporting through practical application (Yükseköğretim Kurulu, 2018). In this context, developing positive attitudes toward scientific research methods is considered important not only for individuals aiming for an academic career but also for all teacher candidates who will work in the field of education (Korkmaz et al., 2011).

It is stated that research experience improves teachers’ research literacy and innovative thinking skills, which in turn positively impacts the quality of teaching (Sun and Chen, 2024). Furthermore, research-based practices are considered to support teachers’ professional development and enrich instructional processes by fostering greater depth (Evans et al., 2017; Li, 2024; Winch et al., 2015). Indeed, teaching is defined as a profession involving evidence-based decision-making processes, and in this context, it is emphasized that research skills are closely related to professional competencies (Everton et al., 2002; Wilson et al., 2012). Accordingly, teacher candidates’ engagement with research methods should not be considered only in terms of course completion or research skill acquisition, but also in relation to their attitudes toward research methods as part of research-oriented teacher preparation.

Learning scientific research processes involves not only the acquisition of cognitive knowledge but also individual attitudes toward these processes. Attitude is defined as a general evaluation of a psychological object, and it is shaped by the interaction of an individual’s beliefs about that object and the values they attribute to those beliefs (Ajzen, 2001). In this context, it is accepted that attitudes are not limited to the cognitive dimension alone, but exhibit a multidimensional structure that also includes affective and behavioral/conative components (Ajzen, 2001; Eagly and Chaiken, 1993; Katz and Stotland, 1959). Since attitudes cannot be directly observed, they can be measured through these indicators. The cognitive component reflects evaluations based on knowledge and beliefs; the affective component reflects feelings and emotional responses; the behavioral component includes intentions and action tendencies (Breckler, 1984; Eagly and Chaiken, 1993; Rosenberg and Hovland, 1960). This multi-component structure of attitude can vary in different contexts, and the relationships between the components can differ depending on the context (Eagly and Chaiken, 1993). Breckler (1984) demonstrated that although the cognitive, affective, and behavioral dimensions of attitude can be distinguished statistically, they remain moderately interrelated. This theoretical perspective provides the basis for examining attitudes toward research methods as a multidimensional construct rather than as a single favorable or unfavorable evaluation.

Studies on attitudes toward educational research show that teachers and teacher candidates exhibit different and sometimes contradictory attitudes toward this field. Previous research has produced mixed findings, with some studies indicating positive attitudes toward educational research among participants (Günyel and Bilgivar, 2023), whereas others have highlighted the predominance of negative attitudes (Butt and Shams, 2013). Furthermore, it has been shown that attitudes toward research in education can differ depending on individual and contextual variables (Murtonen, 2005) and can vary according to factors such as gender (Çelen, 2023). Moreover, studies employing systematic literature reviews have also highlighted the diversity of findings on this issue (Matos et al., 2023). These findings demonstrate that attitudes toward educational research exhibit a complex and multidimensional structure.

Attitudes toward research methods can be considered an important factor influencing students’ participation in research processes, their orientation toward evidence-based practices, and their academic learning experiences in higher education systems. Therefore, examining how such attitudes are structured in different cultural contexts is important both for testing theoretical models and for ensuring the international comparability of the findings.

In the current literature, there are some scales developed to measure the attitudes of teachers and teacher candidates toward research (İlhan et al., 2013; Öztürk, 2011). However, unlike studies conducted to determine general attitudes toward conducting research (Çelik et al., 2012; Korkmaz et al., 2011; Yapalak and Ilgaz, 2013), attitudes toward scientific research practices (Şahin and Avşar, 2013), or attitudes toward the research methods course (Yaşar, 2014), there are a limited number of measurement tools that directly measure attitudes toward the content of research methodology. This situation highlights the need for adapting and validating instruments that directly focus on attitudes toward research methodology content and for examining whether the multidimensional structure of this construct is preserved across different education systems.

Research methods education may also have particular relevance in practice-based teacher education fields such as music education. Preservice music teacher education includes interconnected areas such as musicianship, pedagogical content knowledge, core teaching practices, planning, assessment, field experience, and student teaching (Conway, 2022; Conway et al., 2020). Therefore, examining music teacher candidates’ attitudes toward research methods alongside those of candidates from other teacher education programs may help clarify whether such attitudes represent a shared aspect of teacher preparation across disciplinary contexts.

### 1.1 Purpose and rationale of the study

The main purpose of this study is to adapt the “Attitudes towards Research Methods Questionnaire (ATRMQ)” developed by Matas-Terrón et al. (2024) to Turkish culture and to examine the validity and reliability evidence of the scale. Furthermore, the study aims to validate the factor structure of the scale in the Turkish context. In this respect, the study is not only a scale adaptation study, but also aims to contribute to testing the multidimensional structure of attitudes toward research methods in a different cultural context.

The literature emphasizes that attitude structures can be sensitive to cultural and contextual factors (Farley and Stasson, 2003; Greenwald, 1990; Greenwald and Nosek, 2008). The way research methods education is conducted, the evaluation culture, and academic expectations can differ from country to country. Therefore, retesting the factor structure validated in the original study in a different cultural context is important. Indeed, Matas-Terrón et al. (2024) argue that attitudes toward research methods have received limited comprehensive attention in higher education and that examining them may provide valuable insights into teaching and learning processes.

The scale adapted in this study aims to directly measure attitudes toward the content of research methodology, unlike general attitudes toward conducting research or the course itself. In this respect, the scale reduces the possibility of external influences related to the teaching process (e.g., the way the course is conducted or the instructor factor) being reflected in the measurement, thus offering a more direct opportunity to evaluate teacher candidates’ perceptions of research methods. This approach, differentiating itself from existing measurement tools (Çelik et al., 2012; İlhan et al., 2013; Korkmaz et al., 2011; Öztürk, 2011; Şahin and Avşar, 2013; Yapalak and Ilgaz, 2013; Yaşar, 2014), allows for the examination of attitudes toward research methods in a more specific dimension.

In this context, the aim of the present study is to contribute to the limited number of studies in the teacher education literature that focus on measuring attitudes toward the content of research methodology, and to provide empirical evidence for the development of valid measurement tools in different cultural contexts. In addition, the study examined whether music teacher candidates differed from candidates in other teacher education programs in their overall and dimension-specific attitudes toward research methods.

## 2 Method

### 2.1 Research design

This study was designed as a methodological study focusing on scale adaptation and the examination of psychometric properties. This design was appropriate for the study because it focused on adapting an existing measurement tool into Turkish and examining its psychometric properties in a new cultural context. In the study, the “Attitudes towards Research Methods Questionnaire (ATRMQ)” was adapted into Turkish, and validity and reliability analyses were conducted within the Turkish cultural context. The research was designed and conducted within the framework of quantitative research methods.

### 2.2 Participants

To determine the participants of the study, an online survey link was sent to all universities in Türkiye with official letters after obtaining the necessary permissions. A total of 553 teacher candidates from the faculties of education of various universities voluntarily participated in the study. In Türkiye, the “Research Methods in Education” course is given in the second year at the undergraduate level. Therefore, participants who had previously taken this course were included in the research. In this context, it was ensured that all participants were familiar with research methods topics. Therefore, 44 (7.96%) of the collected data were excluded from the analyses because the participants were in their first or second year. As a result, the initial eligible dataset consisted of 509 cases. The participants consisted of teacher candidates studying in different departments of the faculties of education. Table 1 shows demographic details of the participants.

**Table 1:** Demographic characteristics of the participants.

Demographic variables	*N =* 509	% of sample
Gender		
Female	362	71.1
Male	147	28.9
Grade level		
3rd year	260	51.1
4th year	249	48.9
Department		
Music education	114	22.4
Primary education	93	18.3
English language education	74	14.5
Early childhood education	57	11.2
Social studies education	46	9
Guidance and psychological counseling	28	5.5
Mathematics education	22	4.3
Science education	20	3.9
Art education	19	3.7
Turkish language education	16	3.1
Special education	8	1.6
Computer and instructional technologies education	6	1.2
German language education	6	1.2

Among the disciplinary groups in the eligible sample, music education constituted the largest subgroup (*n* = 114, 22.4%; see Table 1).

### 2.3 Data collection instruments

In this research, an online survey form consisting of three sections was used as a data collection instrument.

#### 2.3.1 Demographic information form

The first section of the survey included questions aimed at determining the personal and academic characteristics of the participants. In this context, demographic variables such as university, department, gender, and year of study were collected.

#### 2.3.2 Turkish form of the attitudes towards research methods questionnaire

The second section of the survey included the adapted Turkish form of the scale, titled “Araştırma Yöntemlerine Yönelik Tutum Ölçeği (AYYTÖ).” The adaptation was based on the instrument developed by Matas-Terrón et al. (2024) to assess students’ attitudes toward research methods. High scores on the scale indicate more positive attitudes toward research methods; low scores indicate more negative attitudes. The scale, consisting of a total of 16 items, covers three sub-dimensions: Negative Emotions, Utility, and Positive Emotions. Items 1, 4, 6, 9, 10, 11, 13, 14, 15, and 16 on the scale are reverse-coded.

In the original development study, the ATRMQ was administered to 447 education students and its psychometric properties were examined through exploratory and confirmatory factor analyses (Matas-Terrón et al., 2024). The exploratory analysis supported a 16-item three-factor structure consisting of Negative Emotions, Positive Emotions, and Utility, explaining 50.70% of the total variance. In the confirmatory stage, alternative models were tested, including models with and without the ambiguous item reported in the original study. The model without this item showed acceptable fit values (*χ*^2^/df = 1.793, CFI = 0.944, TLI = 0.933, SRMR = 0.058, RMSEA = 0.066). The reliability coefficients reported in the original study were also acceptable, with Cronbach’s alpha values ranging from 0.752 to 0.897 and McDonald’s omega values ranging from 0.754 to 0.898.

In the original scale, responses were collected using a 5-point Likert-type scale (1 = Strongly disagree, 5 = Strongly agree), with an additional “I do not know” option coded as 6. The purpose of the sixth option is to distinguish lack of information from the neutral response category (Matas-Terrón et al., 2024). However, there are debates in the literature regarding the interpretation of options such as ‘undecided’, ‘do not know’, and ‘no opinion’, and it is emphasized that such options can create ambiguity in the response process. For example, participants sometimes use such options to indicate neutrality and sometimes to indicate a lack of information (Armstrong, 1987; Baka et al., 2012; Raaijmakers, 2000). It has also been reported that ‘do not know’ responses are relatively rare and do not function as a substitute for the ‘neutral’ option (Mariano et al., 2024). Therefore, in the present study, this option was removed due to its potential to create ambiguity. Accordingly, the scale was administered using a 5-point Likert-type scale, encouraging participants to express more clearly positive or negative attitudes.

#### 2.3.3 Anxiety toward research scale (ATRS)

The third section of the survey included the Anxiety toward Research Scale (ATRS), developed by Büyüköztürk (1997). This scale, designed to determine students’ anxiety levels toward conducting research, consists of 12 items. Having a single-factor structure, the scale is scored on a 5-point Likert-type scale. High scores indicate a high level of research anxiety. The scale accounted for 41.9% of the variance, and its Cronbach’s alpha coefficient for internal consistency was reported as 0.87. Furthermore, the scale showed significant correlations with other scales measuring similar constructs. In this study, ATRS was used for criterion validity purposes.

### 2.4 Translation and adaptation process

The adaptation process of the scale was carried out in accordance with the principles stated by Kılıç et al. (2023). First, the original English items were translated into Turkish by the researchers. The translations were evaluated by two independent language experts, both holding doctoral degrees: one a university lecturer with 24 years of experience, and the other with 12 years of experience. Necessary corrections were made in line with the experts’ opinions, and statistical analysis revealed a high level of agreement between the opinions (*Kendall’s W* = 0.914; *p* = 0.026).

In the next stage, the Turkish items were translated back into English by two different language experts. One of these experts was a university lecturer with 20 years of experience, and the other was an English teacher with 10 years of experience. As a result of the comparisons, no difference in meaning was found between the Turkish and original items.

The Turkish items were also reviewed by three Turkish language experts (with 31, 22, and 15 years of experience) and final linguistic checks were performed. As a result of these stages, the Turkish draft form of the scale was prepared for linguistic equivalence testing. Subsequently, the English and Turkish versions of the scale were administered to 32 fourth-year students in the Department of English Language Teaching at one-week intervals. This group was selected because fourth-year English Language Teaching students were expected to have sufficient proficiency to respond to both the English and Turkish forms and to evaluate the semantic correspondence between the two versions. Because this linguistic equivalence study was conducted before the main factor analytic stage, the analyses were based on the 16-item draft form administered at that stage. The sub-dimension scores were calculated according to the original scoring structure, and these analyses were used to examine cross-language consistency of the corresponding item sets, not to test the factor structure of the Turkish form. Since the difference score for the Utility dimension did not meet the normality assumption according to the *Shapiro–Wilk test*, *Spearman correlation coefficients* and *Wilcoxon signed-rank tests* were used. The English and Turkish forms showed statistically significant positive correlations for the total score (*ρ* = 0.72, *p* < 0.001), Negative Emotions (*ρ* = 0.73, *p* < 0.001), Positive Emotions (*ρ* = 0.62, *p* < 0.001), and Utility (*ρ* = 0.57, *p* < 0.001). Wilcoxon signed-rank tests showed no statistically significant differences between the English and Turkish forms for the total score (*Z* = –1.81, *p* = 0.07), Negative Emotions (*Z* = –0.25, *p* = 0.80), and Positive Emotions (*Z* = –1.83, *p* = 0.07). However, a statistically significant difference was observed for Utility (*Z* = –2.54, *p* = 0.01). Therefore, the linguistic equivalence evidence for the Utility dimension was interpreted cautiously and considered during the final wording review of the Turkish form. Overall, these findings provided preliminary evidence for the semantic equivalence of the Turkish draft form, while indicating that the linguistic equivalence evidence for the Utility dimension required more cautious interpretation.

Following the testing of linguistic equivalence, the Turkish form of the scale was administered to 96 fourth-year students studying in different departments of the education faculty at a state university. This second pilot administration was conducted to examine the comprehensibility, cultural appropriateness, and preliminary item functioning of the Turkish draft form in a group similar to the target population of the main study. In this second pilot study, the participants had similar characteristics to the main sample. Descriptive statistics of the scores obtained from the scale were examined, item-total correlations were calculated, and Cronbach’s alpha coefficient was evaluated. The findings indicated that the Turkish form was clear and understandable for the target audience. However, as a result of these analyses, and after obtaining the opinions of language experts, only minor wording changes were made in some items, and the scale was finalized for the main study.

### 2.5 Procedures

The research data were collected at the end of the spring semester of 2025. The study was approved by the ethics committee of a state university in Türkiye (decision number 2024/429). The survey link was sent to other universities in Türkiye as an attachment to the university’s official permission letter and shared within the institution through faculty members. Only 3rd and 4th year teacher candidates were asked to answer the survey.

The online survey was administered via Google Form, and since all questions were set as mandatory fields, there was no data loss. All participants voluntarily completed the survey after reading and approving the informed consent text at the beginning of the survey.

### 2.6 Data analysis

SPSS 27 and AMOS 22 software were used in the analysis of the data. In the linguistic equivalence stage, the relationships between the English and Turkish forms were examined using *Spearman correlation* coefficients. Since the difference score for the Utility dimension did not meet the normality assumption, *Wilcoxon signed-rank tests* were used to examine mean differences between the English and Turkish forms.

First, a missing data check was performed, and it was determined that there was no data loss. The reverse-coded items in the scale (items 1, 4, 6, 9, 10, 11, 13, 14, 15, and 16) were appropriately recoded before analysis. Descriptive statistics were used to examine participants’ demographic characteristics, as well as mean, standard deviation, skewness, and kurtosis values.

Multivariate outliers were identified by examining *Mahalanobis distance* values, and a *p* < 0.001 level was considered for the *χ*^2^ value in the evaluations (Hair et al., 2019; Tabachnick and Fidell, 2013). Multivariate normality distribution was tested with *Mardia’s coefficient*, and the critical value was calculated using the *p*(*p* + 2) formula suggested in the literature (Bollen, 1989; Raykov and Marcoulides, 2008). Here, *p* represents the number of observed variables. Scale scores for univariate outliers were converted to *Z-*scores, and data outside the ±3 threshold were checked (Raykov and Marcoulides, 2008). *Skewness* and *kurtosis* values were examined to assess univariate normality, and values within the ±1.5 range were considered acceptable (Schumacker and Lomax, 2004).

After data screening, the final dataset of 486 cases was randomly divided into two independent subsamples. Exploratory factor analysis was conducted on the first subsample (*n* = 243), and confirmatory factor analysis was conducted on the second subsample (*n* = 243). This split-half approach was used to reduce the risk of capitalization on chance and to provide cross-validation evidence for the factor structure.

To measure the suitability of the data set for factor analysis, *KMO and Bartlett’s Test* results were examined (Kılıç et al., 2023). In exploratory factor analysis, the *Principal Axis Factoring (PAF)* method was preferred as the factor extraction technique. PAF, recommended by experts in scale development and adaptation studies in the literature, reveals latent structures more accurately by considering the common variance of the observed variables (Furr, 2022). As a rotation technique, the *Direct Oblimin* method, which considers the relationships between factors, was used (Field, 2024). In the analyses, a lower limit of 0.30 was considered for factor loadings, and this value is recommended as the minimum threshold accepted in factor analyses in the literature (Field, 2024). In determining the number of factors, eigenvalues greater than 1, the total variance explained, and the elbow point in the scree plot were considered (Field, 2024; Kılıç et al., 2023). Items were evaluated in terms of factor loadings and cross-loadings. The item that did not show a clear salient loading on a single factor was further examined by testing alternative CFA models with and without the item.

Confirmatory factor analysis was performed using the *Maximum Likelihood (ML)* method since the assumption of multivariate normality was met (Kline, 2016; Raykov and Marcoulides, 2008). The model’s fit was assessed using commonly reported goodness-of-fit indices (*CMIN/DF (χ^2^/df)*, *RMSEA*, *SRMR*, *CFI*, *TLI, IFI, NFI, AGFI,* and *GFI*). These values were interpreted according to the threshold levels suggested in the literature and presented in Table 2 (Byrne, 2016; Hu and Bentler, 1999; Kline, 2016; Schumacker and Lomax, 2004).

**Table 2:** Goodness of fit values.

Fit indices	Good fit	Acceptable
CMIN/DF	0 ≤ χ^2^/df≤3	3 < χ^2^/df<5
RMSEA	0 ≤ RMSEA≤0.05	0.05 < RMSEA≤0.08
SRMR	0 ≤ SRMR≤0.05	0.05 < SRMR≤0.08
CFI	0.95 ≤ CFI<1.00	0.90 ≤ CFI<0.95
TLI	0.95 ≤ TLI<1.00	0.90 ≤ TLI<0.95
IFI	0.95 ≤ IFI<1.00	0.90 ≤ IFI<0.95
NFI	0.95 ≤ NFI<1.00	0.90 ≤ NFI<0.95
AGFI	0.95 ≤ AGFI<1.00	0.90 ≤ AGFI<0.95
GFI	0.95 ≤ GFI<1.00	0.90 ≤ GFI<0.95

Reliability analyses were performed for the scale and its sub-dimensions, and *Cronbach’s alpha* coefficients were calculated (Field, 2024). *McDonald’s omega* coefficients were also calculated to provide additional evidence for internal consistency (Kalkbrenner, 2024). In addition, *Composite Reliability (CR)* and *Average* Var*iance Extracted (AVE)* values were calculated using the standardized factor loadings obtained from the CFA (Hair et al., 2019). In addition, to evaluate item discrimination, *corrected item-total correlations* were examined, and the difference between the scores of the lower and upper 27% groups were tested with an *Independent Samples t-test* (Erkuş, 2019). Effect sizes for item discrimination were reported using *Cohen’s d* (Cohen, 1988).

Correlation analyses with a criterion measure were performed within the scope of c*riterion validity* (Kılıç et al., 2023). Before the criterion validity analysis, the reliability coefficient of the criterion scale was calculated, and the normality of the criterion scale scores was examined. Since the normality assumption was met, Pearson correlation coefficients were used to examine the relationships between the AYYTÖ total and sub-dimension scores and the criterion measure.

In addition, to examine discipline-based differences, music teacher candidates were compared with candidates enrolled in other teacher education programs. *Welch’s independent-samples t-test* was used for the total and subscale scores because of the unequal group sizes (Field, 2024). *Holm-adjusted p values* were considered for multiple comparisons, and effect sizes were reported using *Hedges’ g* (Holm, 1979; Lakens, 2013).

## 3 Results

### 3.1 Assessment of normality and outliers

Multivariate outliers were determined by examining *Mahalanobis distance* coefficients, and 16 responses (3.14%) that were above the critical value at the *p* < 0.001 level for the *χ^2^* distribution were excluded from the analysis (Tabachnick and Fidell, 2013). Subsequently, Mardia’s multivariate kurtosis coefficient was calculated to determine whether multivariate normality was achieved. The obtained value was 57.46, which is below the critical value of 288 calculated using the *p*(*p* + 2) formula for a model with 16 observed variables. Accordingly, it can be said that the assumption of multivariate normality is met (Bollen, 1989; Raykov and Marcoulides, 2008).

To identify univariate outliers, scale scores were converted to *Z*-scores, and 7 responses (1.42%) outside the ±3 threshold in the remaining dataset of 493 participants were excluded from the analysis (Raykov and Marcoulides, 2008). As a result, a total of 23 participants (4.52%) were excluded from the analysis, and the analyses were conducted on the remaining 486 participants. Next, skewness and kurtosis values were examined to determine whether univariate normality was achieved.

Table 3 presents descriptive statistics reflecting item-level distributions before factor analyses. Accordingly, the skewness values of the items range from −1.15 to 0.11, and the kurtosis values range from −0.71 to 1.23. The fact that all values are within the ±1.5 range (mostly close to zero except for one item) indicates that the assumption of univariate normality is met (Schumacker and Lomax, 2004). On the other hand, the median values of the items range from 3 to 4. These findings support the symmetry of the item-level distributions. Thus, the normality assumptions were met in the dataset of 486 responses.

**Table 3:** Descriptive statistics for variables.

Variables	Median	Mean	SD	Skewness	Kurtosis
M1	3	3.31	0.83	−0.29	0.12
M2	3	3.42	0.89	−0.38	0.10
M3	3	3.29	0.98	−0.37	−0.32
M4	3	2.92	0.86	0.06	−0.59
M5	3	3.27	0.96	−0.36	−0.29
M6	3	3.11	0.99	−0.22	−0.55
M7	3	2.90	0.92	0.11	−0.64
M8	3	3.01	1.08	−0.03	−0.71
M9	4	3.39	0.94	−0.40	−0.40
M10	4	4.30	0.81	−1.15	1.23
M11	3	2.92	0.97	−0.04	−0.40
M12	4	3.93	0.80	−0.46	0.08
M13	4	4.03	0.78	−0.64	0.39
M14	4	3.51	0.91	−0.52	0.02
M15	4	3.88	0.88	−0.74	0.41
M16	4	3.51	0.95	−0.36	−0.44

### 3.2 Exploratory factor analysis (EFA)

Exploratory factor analysis was conducted on the first randomly selected subsample (*n* = 243). Before factor analysis, the suitability of the data for factor analysis was tested. The *Kaiser-Meyer-Olkin (KMO)* value was 0.91, which is classified as *“marvelous”* according to the literature, as values above 0.90 fall within this category (Field, 2024). *Bartlett’s Test of Sphericity* was significant (*χ^2^*(120) = 1717.69, *p* < 0.001). In the anti-image correlation matrix, it was seen that the *Measures of Sampling Adequacy (MSA)* diagonal values ranged from 0.84 to 0.95. In the literature, it is stated that MSA values above 0.50 (preferably higher) indicate sample suitability (Field, 2024).

When the communalities values were examined, it was seen that the extraction values of the items were in the range of 0.35 to 0.73. In the literature, communalities values below 0.20 are reported to indicate low common variance (Child, 2006), and values above 0.30 are considered appropriate (Leech et al., 2015). This finding shows that each item carries common variance with other items. Therefore, all these findings indicate that the dataset is suitable for factor analysis (Kılıç et al., 2023).

**Figure 1:**
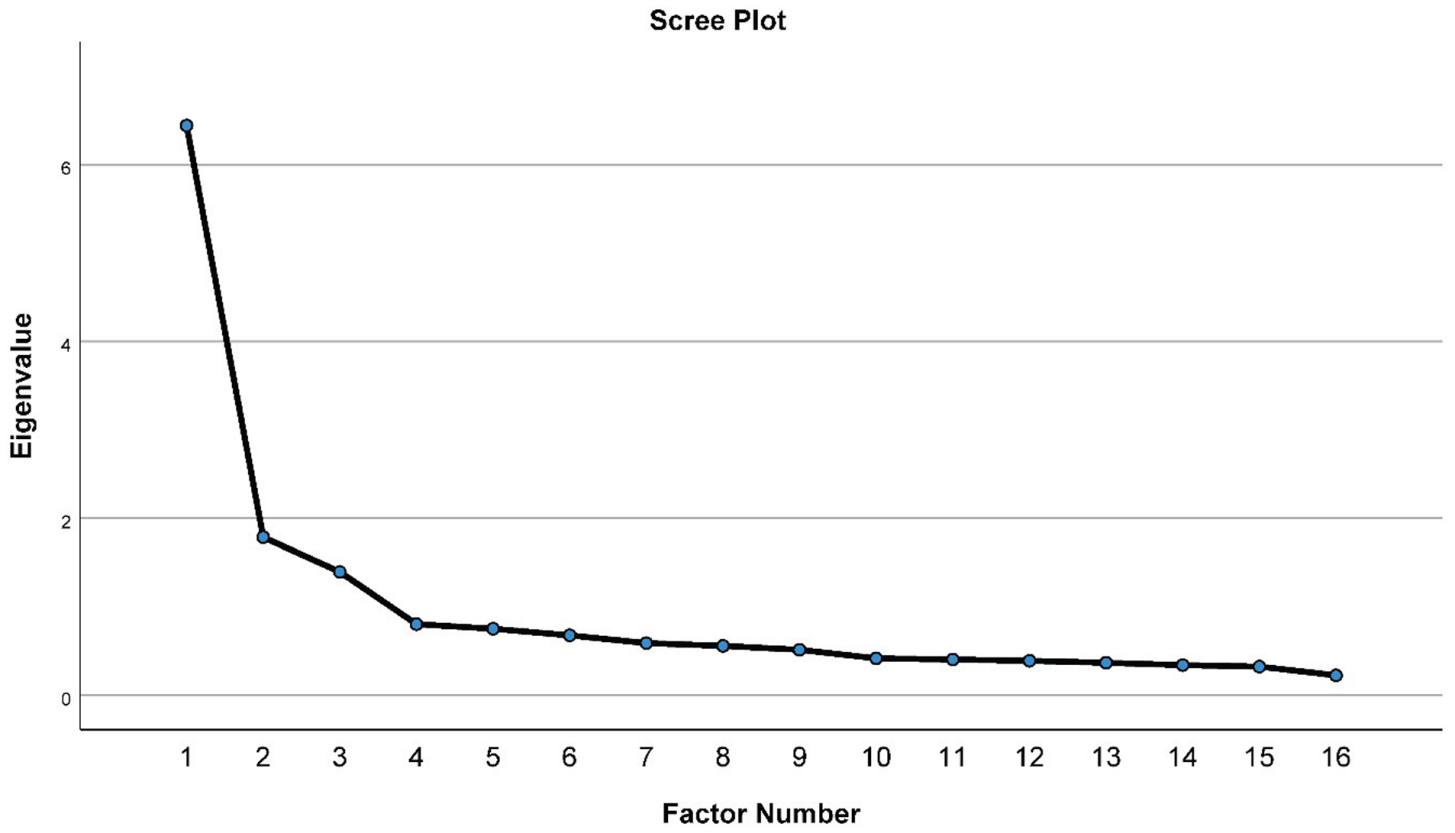
Scree plot.

As a result of EFA performed using the Principal Axis Factoring (PAF) method and Direct Oblimin rotation, three factors with an initial eigenvalue above 1 were obtained. Together, the three factors explain 51.55% of the common variance, as presented in Table 4. The Scree Plot presented in Figure 1 also supports a three-factor structure (Field, 2024; Kılıç et al., 2023).

**Table 4:** Eigenvalues and explained variance.

Factor	Initial eigenvalues	% of variance (extraction)	Cumulative % (extraction)
1	6.44	37.41	37.41
2	1.79	7.94	45.34
3	1.40	6.21	51.55

Extraction method: Principal axis factoring (PAF); Rotation: Direct oblimin.

According to the Pattern Matrix results in Table 5, M1, M4, M6, M9, M11, M14, and M16 loaded onto the first factor; M10, M12, M13, and M15 loaded onto the second factor; and M2, M3, M5, and M8 loaded onto the third factor. Except for M7, the items generally had factor loadings around or above 0.40 (Field, 2024), and the factor loadings showed a clear three-factor pattern. However, M7 did not show a salient loading on a single factor and produced comparable loadings on the first factor (0.37) and the third factor (0.39). Howard (2016) notes that there is no consensus in the literature regarding the conditions under which an item loading onto more than one factor should be considered cross-loading, with some studies considering the difference between factor loadings as 0.20 and others as 0.10. In the present study, the difference between the two loadings for M7 was very small, and the item did not reach a clear salient loading on either factor. Therefore, M7 was identified as an overlapping item. To examine the effect of this item on the measurement model, two alternative models were tested in the independent CFA subsample: one model including M7 and another model excluding M7.

**Table 5:** Pattern matrix.

Item	Factor 1	Factor 2	Factor 3
M9	0.80		
M14	0.75		
M6	0.75		
M16	0.70		
M4	0.61		
M1	0.49		
M11	0.40		
M12		0.70	
M13		0.68	
M10		0.62	
M15		0.55	
M5			0.81
M2			0.78
M3			0.67
M8			0.56
M7	0.37		0.39

Extraction method: Principal axis factoring. Rotation method: Direct oblimin.

Loadings <0.30 are suppressed.

Examination of the Structure Matrix and Factor Correlation Matrix revealed moderate positive correlations among the factors, supporting the use of Direct Oblimin rotation. The factor correlations ranged from 0.30 to 0.58. As a result of the analyses, a three-factor structure was obtained, as in the original scale. The first factor was named “Negative Emotions,” the second factor “Utility,” and the third factor “Positive Emotions,” in parallel with the original scale.

### 3.3 Confirmatory factor analysis (CFA)

The three-factor structure obtained as a result of EFA was tested with CFA using AMOS software on the second randomly selected subsample (*n* = 243). Since the assumption of multivariate normality was met, the *Maximum Likelihood (ML)* method was used in parameter estimation.

To evaluate the effect of M7, which showed an overlapping loading pattern in the EFA, within the CFA framework, two alternative models were tested in the independent CFA subsample. Similarly, it was reported that different models were tested for the corresponding ambiguous item in the original scale development study (Matas-Terrón et al., 2024). Accordingly, Model 1, which included M7, was tested first, followed by Model 2, in which M7 was excluded. The fit indices for both models are presented in Table 6.

**Table 6:** Fit indices for confirmatory factor analyses.

Model	*χ^2^/df*	RMSEA	SRMR	CFI	TLI	IFI	NFI	AGFI	GFI
Model 1	2.43	0.08	0.07	0.92	0.90	0.92	0.87	0.85	0.89
Model 2	2.07	0.07	0.06	0.94	0.93	0.94	0.90	0.87	0.91

**Figure 2:**
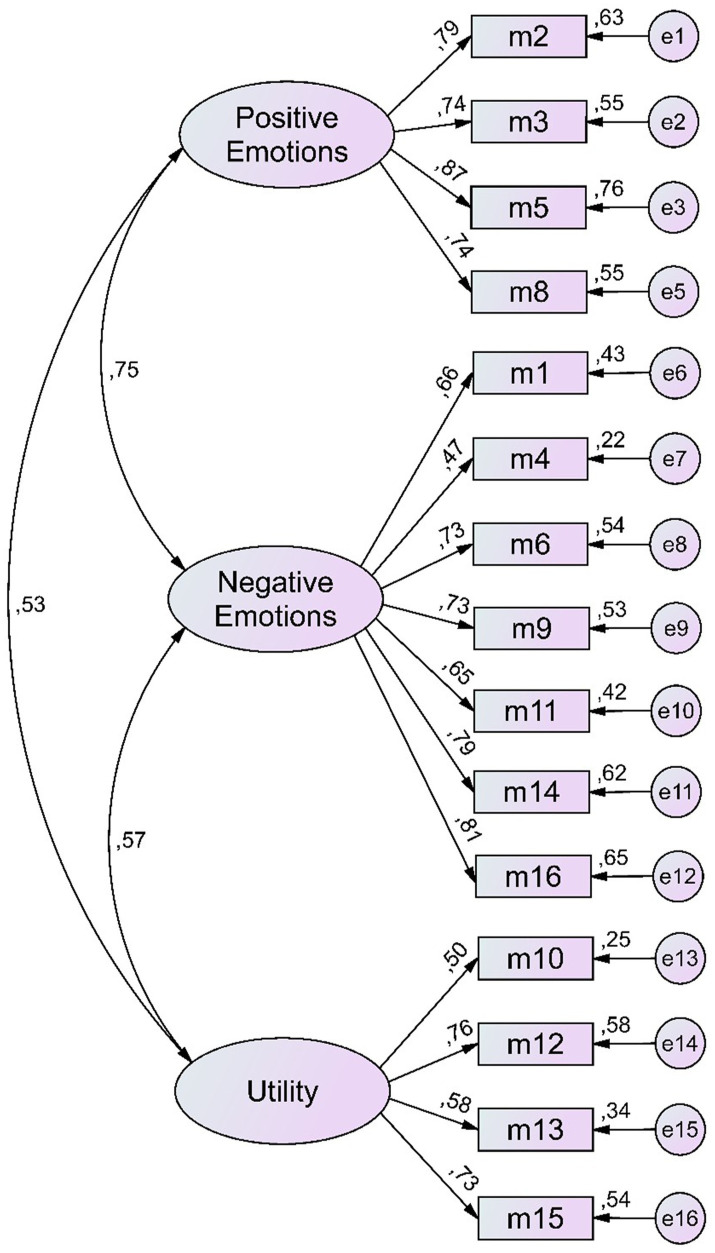
Standardized CFA model.

Compared to Model 1, Model 2 showed better values in all fit indices (see Table 6). This improvement observed in Model 2, when compared with the suggested threshold values of the fit indices (see Table 2), indicates that the overlapping structure of M7 has an effect on model fit. Although AGFI remained slightly below the conventional 0.90 threshold, the overall pattern of fit indices supported an acceptable model fit. The standardized path diagram for Model 2 is presented in Figure 2.

The standardized factor loadings of all items in Model 2 range from 0.47 to 0.87 and are all statistically significant (*p* < 0.001). Although one item had a relatively modest standardized loading (0.47), all standardized loadings were statistically significant, and the overall pattern of loadings supported the three-factor structure. This finding indicates that the items adequately represent the relevant latent constructs. The correlations between latent variables ranged from 0.53 to 0.75, indicating moderate to high relationships among the factors and showing that the factors were related but distinguishable constructs (Hair et al., 2019; Kline, 2016). To maintain theoretical integrity, no modifications or error covariances suggested by modification indices were added to the model. These results support the three-factor measurement model.

### 3.4 Reliability and item analyses

The reliability of the scale was evaluated using *Cronbach’s alpha* coefficient and *McDonald’s omega* coefficient (Field, 2024; Kalkbrenner, 2024). The analyses were performed on the final 15-item form obtained from the CFA results. Accordingly, the total Cronbach’s alpha coefficient of the scale was found to be 0.90, indicating a high level of internal consistency. The total McDonald’s omega coefficient was found to be 0.90, supporting the reliability evidence obtained from Cronbach’s alpha. Furthermore, it was observed that removing any item did not lead to a significant increase in the Cronbach’s alpha coefficient. These findings demonstrate the strong internal consistency of the scale. Cronbach’s alpha, McDonald’s omega, Composite Reliability (CR), and Average Variance Extracted (AVE) values for the scale and its sub-dimensions are presented in Table 7.

**Table 7:** Reliability coefficients and construct reliability indices of the scale.

Dimension	Cronbach’s α	McDonald’s ω	CR	AVE
Negative Emotions	0.87	0.87	0.87	0.49
Positive Emotions	0.85	0.85	0.87	0.62
Utility	0.76	0.76	0.74	0.42
Total Scale	0.90	0.90	—	—

Cronbach’s alpha and McDonald’s omega were calculated using the final 486-case dataset. CR and AVE were calculated using the standardized factor loadings obtained from the independent CFA subsample.

McDonald’s omega coefficients were consistent with Cronbach’s alpha coefficients, ranging from 0.76 to 0.90. CR values ranged from 0.74 to 0.87, indicating acceptable construct reliability (Hair et al., 2019). The AVE value was above 0.50 for Positive Emotions and close to the 0.50 threshold for Negative Emotions, whereas the Utility dimension showed a lower AVE value (Hair et al., 2019). Therefore, the AVE results were interpreted together with CR values, standardized factor loadings, and internal consistency coefficients.

Corrected item–total correlations ranged from 0.34 to 0.71. Item–total correlations of all items above 0.30 indicate that the items represent the construct measured by the scale in a distinctive manner (Field, 2024). Item-level corrected item-total correlations and discrimination statistics are presented in Table 8.

**Table 8:** Item analysis results.

Item	Corrected item-total correlation	Upper 27% *M* (SD)	Lower 27% *M* (SD)	*t*	*p*	Cohen’s *d*
M1	0.60	3.99 (0.65)	2.69 (0.75)	15.11	*<0.001*	1.87
M2	0.69	4.16 (0.58)	2.59 (0.81)	18.04	*<0.001*	2.23
M3	0.54	3.98 (0.74)	2.55 (0.85)	14.52	*<0.001*	1.79
M4	0.43	3.45 (0.77)	2.45 (0.76)	10.62	*<0.001*	1.31
M5	0.68	4.07 (0.65)	2.40 (0.83)	18.10	*<0.001*	2.24
M6	0.62	4.01 (0.63)	2.34 (0.77)	19.16	*<0.001*	2.37
M8	0.62	3.92 (0.80)	2.14 (0.87)	17.34	*<0.001*	2.14
M9	0.67	4.15 (0.60)	2.54 (0.74)	19.39	*<0.001*	2.40
M10	0.34	4.72 (0.45)	3.79 (0.93)	10.19	*<0.001*	1.26
M11	0.56	3.60 (0.79)	2.15 (0.80)	14.77	*<0.001*	1.82
M12	0.51	4.50 (0.53)	3.38 (0.77)	13.74	*<0.001*	1.70
M13	0.40	4.47 (0.66)	3.54 (0.79)	10.29	*<0.001*	1.27
M14	0.66	4.22 (0.57)	2.66 (0.81)	18.04	*<0.001*	2.23
M15	0.49	4.48 (0.65)	3.19 (0.84)	13.89	*<0.001*	1.72
M16	0.71	4.34 (0.55)	2.57 (0.77)	21.25	*<0.001*	2.63

Upper and lower groups were formed based on the total score of the final 15-item scale. Each group included 27% of the final sample (*n* = 131 per group). Italicized values indicate statistical significance at *p* < 0.001.

Item discrimination was examined by comparing the item scores of the lower 27% and upper 27% groups using the *Independent Samples t-test*. According to the analysis results, statistically significant differences were found between the lower and upper groups for all items (Erkuş, 2019). The obtained effect size values ranged from 1.26 to 2.63, indicating that the discrimination levels of the items are high. Considering that a *Cohen’s d* value above 0.80 is evaluated as a large effect size (Cohen, 1988), these findings reveal that all items of the scale have strong discriminatory power.

### 3.5 Criterion validity

Criterion validity was examined by correlation analysis with the Anxiety toward Research Scale (ATRS) developed by Büyüköztürk (1997). In the present study, the *Cronbach’s alpha* coefficient of the ATRS was 0.93. Before the analysis, the normality assumption of the ATRS total scores was evaluated through skewness (0.40) and kurtosis (0.13) values. Since these values were within the acceptable range, the normality assumption was considered to be met (Schumacker and Lomax, 2004). Accordingly, the relationship between the AYYTÖ total and sub-dimension scores and the ATRS total score were analyzed using *Pearson correlation* coefficients (Field, 2024).

As shown in Table 9, the findings showed negative and statistically significant correlations between the ATRS total score and all AYYTÖ scores. The strongest correlation was found between the AYYTÖ total score and the ATRS total score (*r* = −0.73, *p* < 0.001). At the sub-dimension level, the ATRS total score was negatively correlated with Negative Emotions (*r* = −0.66, *p* < 0.001), Positive Emotions (*r* = −0.62, *p* < 0.001), and Utility (*r* = −0.47, *p* < 0.001). These findings indicate that more positive attitudes toward research methods are associated with lower research anxiety scores, thereby supporting the criterion validity of the scale.

**Table 9:** Correlations between AYYTÖ scores and research anxiety.

AYYTÖ score	ATRS total score *r*	*p*
Negative Emotions	−0.66	<0.001
Positive Emotions	−0.62	<0.001
Utility	−0.47	<0.001
Total score	−0.73	<0.001

### 3.6 Participants’ attitudes toward research methods

The average total score obtained by the participants from the final form of the AYYTÖ (15 items) was calculated as 51.81 (*SD =* 8.73). The skewness (−0.24) and kurtosis (0.15) values of the total scores indicate that the scores are normally distributed (Schumacker and Lomax, 2004). This descriptive information was reported to provide a general overview of the final scale scores before the discipline-based group comparison.

### 3.7 Comparison of music teacher candidates and other teacher candidates

Following the descriptive overview of the final scale scores, music teacher candidates were compared with candidates enrolled in other teacher education programs to examine discipline-based differences. This comparison was conducted because music education represented the largest disciplinary subgroup in the sample and because it offered a relevant context for examining the use of the adapted scale across teacher education disciplines. As shown in Table 10, *Welch’s independent-samples t-test* results indicated no statistically significant differences between the two groups in Negative Emotions, Utility, Positive Emotions, or total scale scores after *Holm correction* (Field, 2024; Holm, 1979). *Hedges’ g* values ranged from −0.02 to 0.16, indicating that the group differences were close to zero across all comparisons (Lakens, 2013).

**Table 10:** Comparison of attitudes toward research methods between music teacher candidates and other teacher candidates.

Dimension	Music teacher candidates *n* = 108 *M* (SD)	Other teacher candidates *n* = 378 *M* (SD)	Welch’s *t*	*df*	Holm-adjusted *p*	Hedges’ *g*
Negative emotions	23.27 (4.89)	22.50 (4.77)	1.44	169.47	0.604	0.16
Utility	16.11 (2.51)	16.16 (2.49)	−0.17	171.95	0.998	−0.02
Positive emotions	13.19 (3.45)	12.93 (3.21)	0.68	163.58	0.998	0.08
Total score	52.56 (9.20)	51.60 (8.60)	0.98	164.24	0.984	0.11

## 4 Discussion

In this study, the psychometric properties of the Turkish form of the Attitudes toward Research Methods Scale in Education (AYYTÖ) were examined. The findings show that the scale can be used as a valid and reliable measurement tool in a sample of Turkish pre-service teachers. The results of exploratory and confirmatory factor analyses confirmed the three-factor structure of the scale, and evidence from reliability, item analysis, and criterion validity further supported this structure.

The fact that the obtained three-factor structure parallels the factor structure in the original study (Matas-Terrón et al., 2024) indicates that the structural integrity of the scale can be preserved in a cross-cultural context. This finding suggests that the multidimensional structure of attitudes toward research methods can emerge similarly in different cultural contexts and provides an important indicator of the comparability of this structure at the cross-cultural level. The findings are consistent with theoretical approaches that emphasize that attitudes are shaped by cognitive, affective, and behavioral processes and exhibit a multi-component structure (Ajzen, 2001; Eagly and Chaiken, 1993; Katz and Stotland, 1959; Rosenberg and Hovland, 1960; Zanna and Rempel, 1988). The fact that the *Utility* dimension reflects cognitive evaluations of research methods, while the *Positive* and *Negative Emotions* dimensions reflect affective responses, demonstrates the conceptual consistency of the resulting structure. Although the scale does not directly measure behavioral intentions, considering that attitude is a significant determinant of behavioral intentions within the framework of Ajzen’s (1991, 2001) Theory of Planned Behavior, the resulting multidimensional structure can be evaluated as an indirect indicator of potential behavioral orientations. Accordingly, it can be said that considering attitudes toward research methods as a multidimensional structure consisting of cognitive and affective components is theoretically meaningful.

The validation of the three-factor structure also provides an empirical contribution to the discussions on the separability of attitude components. The moderate correlations between factors are consistent with approaches suggesting that the cognitive and affective components of attitude are related yet structurally distinct (Breckler, 1984; Eagly and Chaiken, 1993; Rosenberg and Hovland, 1960). However, there are also views suggesting that attitude components may not be clearly separable in every context and that the relationships between these components may be context-sensitive (Farley and Stasson, 2003; Greenwald, 1990; Greenwald and Nosek, 2008). Indeed, in some scale development studies conducted on different samples, it has been observed that attitude components do not always emerge as separate factors, and in some cases, one-dimensional or two-dimensional structures are obtained (de Boer et al., 2012). The findings of the present study suggest that the empirical differentiation of attitude components may be sensitive to the attitude object and research context. This situation indicates that pedagogical variables can also play a significant role in attitudes. For example, pedagogical variables such as course delivery in research methods, assessment approaches, and classroom climate may influence the interaction between cognitive and affective components. In this context, it can be suggested that positive attitudes toward research methods may be related to research intention, academic participation, and research-based learning behaviors. Therefore, examining the effects of different teaching approaches on attitude components offers a meaningful direction for future research. Considering that attitude components may vary depending on contextual conditions (Farley and Stasson, 2003; Greenwald, 1990; Greenwald and Nosek, 2008), the inclusion of the behavioral intention dimension in the model in the future and the joint examination of the effects of different teaching approaches on attitude components may contribute to a more comprehensive consideration of the theoretical framework. Moreover, comparing the current findings with data obtained in different countries and education systems would provide stronger evidence regarding the cross-cultural consistency of the structural characteristics of attitudes.

Considering that research methods, especially in the context of teaching processes, are often perceived by students as complex and anxiety-inducing (Papanastasiou and Zembylas, 2008; Wishkoski et al., 2022), measuring positive and negative emotions as separate dimensions allows for a more detailed assessment of the affective structure of attitudes toward research methods. Furthermore, systematically measuring students’ affective and cognitive evaluations of research methods is also important from an instructional design perspective (Matas-Terrón et al., 2024). In this context, the AYYTÖ offers a functional measurement tool that allows for the evaluation of both students’ emotional responses and their perceptions of usefulness regarding research methods, both together and separately.

During the EFA process, item M7 showed comparable loadings on two factors and did not produce a clear salient loading on a single factor. This indicated that the item had an overlapping loading pattern. Although there is no definitive consensus in the literature regarding the evaluation of cross-loaded or overlapping items (Howard, 2016), it is suggested that such items may limit model fit and should be tested with alternative models (DeVellis, 2017; Kline, 2016). Comparisons made in this direction showed that the model from which item M7 was removed offered better fit values. Similarly, the fact that alternative models for the corresponding ambiguous item were tested in the original scale development study (Matas-Terrón et al., 2024) suggests that this may reflect a structural issue rather than a sample-specific one.

Reliability analyses revealed that the scale has sufficient internal consistency at both the total score and sub-dimension levels. The fact that the total Cronbach’s alpha coefficient was found to be 0.90 indicates that the scale has a high level of internal consistency. The alpha values for the sub-dimensions being above acceptable limits also supports the internal consistency of the factor structure (Field, 2024). Furthermore, the corrected item-total correlation values being above 0.30 for all items (Field, 2024) and the items significantly distinguishing between the upper and lower 27% groups (Cohen, 1988; Erkuş, 2019) shows that the scale also provides item-level measurement evidence.

The criterion-related validity of the scale was examined through its relationship with research anxiety. The negative and statistically significant correlations between the AYYTÖ scores and ATRS total score indicate that more positive attitudes toward research methods are associated with lower research anxiety scores. The strongest relationship was observed between the AYYTÖ total score and research anxiety, while the sub-dimensions also showed negative and statistically significant relationships with research anxiety. This result aligns with studies showing that research methods are frequently perceived as anxiety-provoking and challenging by students (Büyüköztürk, 1997; Czarnecka and Massaro, 2021; Papanastasiou and Zembylas, 2008; Wishkoski et al., 2022). The finding suggests that attitude and anxiety are related but not overlapping constructs. In this context, it can be said that this relationship offers a significant indicator of the structure of affective experiences toward research methods.

Given that the participants’ total mean score (*M* = 51.81; *SD =* 8.73) was above the midpoint (45) of the possible score range (15–75), attitudes toward research methods in the sample appeared to be generally positive. However, the standard deviation (SD) also indicates variation in participants’ scores. This finding suggests that teacher candidates’ attitudes toward research methods are not homogeneous and that individual differences should be considered when interpreting attitudes toward research methods.

The supplementary comparison between music teacher candidates and candidates from other teacher education programs showed no significant differences in overall or dimension-specific attitudes toward research methods. This finding suggests that, in the present sample, music teacher candidates’ attitudes were broadly similar to those of candidates in other teaching fields. From an instructional perspective, research methods education may therefore be considered a shared component of teacher preparation, including practice-based fields such as music education. This finding should therefore be interpreted as supplementary evidence regarding the use of the adapted scale across teacher education disciplines.

The findings offer important implications not only regarding the psychometric properties of the scale but also regarding the design of research methods education. The fact that the cognitive (perception of utility) and affective (positive and negative emotions) components of attitudes toward research methods are separable indicates that one-dimensional interventions may not be sufficient in instructional design. Cognitive strategies that only emphasize the usefulness of research methods may not automatically reduce students’ negative emotional responses; similarly, only emotionally supportive practices may not strengthen the perception of usefulness. This situation highlights the need for holistic approaches in teaching research methods that target both cognitive and affective dimensions. Future studies could test the predictive roles of affective and cognitive attitude components on research self-efficacy, classroom participation, orientation toward research-based practices, and academic performance. Such an approach would allow for positioning research education processes within explanatory models, rather than treating attitude solely as a descriptive variable.

Considering the relationship between attitude and behavioral consequences (Ajzen, 1991, 2001), it is important to consider this construct together with other cognitively based psychological variables that affect learning processes, such as self-efficacy. Self-efficacy is considered a fundamental variable affecting individuals’ effort, perseverance, and performance (Bandura, 1997). It may be expected that students with more positive attitudes toward research methods and higher research self-efficacy would be more inclined to participate actively in research processes. Accordingly, future studies examining the relationships between attitudes toward research methods, self-efficacy, and anxiety using structural equation modeling could contribute to the of models that holistically explain the affective and cognitive dimensions of attitude.

The Turkish form of the Attitudes Toward Research Methods Scale in Education, titled “Araştırma Yöntemlerine Yönelik Tutum Ölçeği (AYYTÖ),” can be found in Appendix 1.

### 4.1 Limitations and recommendations

While the findings reveal that the scale possesses strong psychometric properties, some limitations should be considered. First, this study is based on a Turkish adaptation of a scale developed in a different cultural context, and the data were obtained from a single national context. Retesting the scale in different cultural, academic, and disciplinary contexts will strengthen the evidence regarding structural validity and generalizability. Second, the data were collected through self-report, which limits the interpretation of the measurements as purely objective assessments. In self-report-based measurements, participants’ tendencies to respond in a more socially acceptable manner can influence their response patterns (Paulhus, 1991; Podsakoff et al., 2003). This possibility should not be entirely ruled out in the present study. Third, the cross-sectional design of the study limits the drawing of conclusions regarding the change in attitudes over time. Although EFA and CFA were conducted on independent randomly divided subsamples in the present study, additional validation studies with larger and more diverse independent samples would provide stronger evidence for the stability of the factor structure. Finally, measurement invariance across subgroups such as gender, grade level, and disciplinary program was not tested. Future studies should examine configural, metric, and scalar invariance to determine whether the scale functions equivalently across different groups of teacher candidates.

## 5 Conclusion

In conclusion, the findings indicate that the Turkish form (AYYTÖ) is a valid and reliable measurement tool for assessing attitudes toward research methods within Turkish teacher education contexts. The scale can be used to examine pre- and post-course changes, evaluate research training programs, and investigate the relationships between attitudes and variables such as self-efficacy, research anxiety, and academic performance. In particular, examining how attitudes toward research methods predict participation in research-based practices in teacher training programs and discipline-based fields, such as music education, offers an important research direction that can be empirically tested in the future.

## Data Availability

The raw data supporting the conclusions of this article will be made available by the authors, without undue reservation.

## Appendix

**Table A1:** Turkish form of the Attitudes Toward Research Methods Scale in Education.

Araştirma Yöntemlerine Yönelik Tutum Ölçeği (AYYTÖ)	Kesinlikle katılmıyorum	Katılmıyorum	Kısmen katılıyorum	Katılıyorum	Kesinlikle katılıyorum
Araştırma yöntemlerinde iyi değilim	①	②	③	④	⑤
Araştırma yöntemlerini kullanmak zevklidir	①	②	③	④	⑤
Araştırma yöntemlerini başkalarıyla tartışmaktan hoşlanırım	①	②	③	④	⑤
Araştırma yöntemleriyle ilgili bir sorunla karşılaştığımda net düşünemediğimi hissediyorum	①	②	③	④	⑤
Araştırma yöntemleri keyifli ve ilgi uyandırıcıdır	①	②	③	④	⑤
Araştırma yöntemleriyle çalışırken kendimi gergin hissediyorum	①	②	③	④	⑤
Araştırma yöntemlerini kullanmayı gerektiren bir mesleğim olsun isterim	①	②	③	④	⑤
Araştırma yöntemleri problemleri üzerinde çalışırken kendimi güvensiz hissediyorum	①	②	③	④	⑤
İstatistikler işe yaramazdır/gereksizdir	①	②	③	④	⑤
Araştırma yöntemleri karmaşık konulardır	①	②	③	④	⑤
Araştırma yöntemleri mesleki eğitimim için bir gerekliliktir	①	②	③	④	⑤
Araştırma yöntemleri çoğu meslek için faydalı değildir	①	②	③	④	⑤
Araştırma yöntemleri analizlerini yaparken kendimi çaresiz hissediyorum	①	②	③	④	⑤
Araştırma yöntemlerini mesleğimde kullanmayacağım	①	②	③	④	⑤
Araştırma yöntemlerinden çekiniyorum	①	②	③	④	⑤
